# Reduced bone mineral density in young adults following cure of acute lympblastic leukaemia in childhood

**DOI:** 10.1038/sj.bjc.6690296

**Published:** 1999-04

**Authors:** B M D Brennan, A Rahim, J A Adams, O B Eden, S M Shalet

**Affiliations:** Department of Paediatric Oncology, Royal Manchester Children's Hospital, Hospital Road, Manchester, M27 4HA, UK; Department of Radiology, University of Manchester, Oxford Road, Manchester, M13 9PT, UK; Department of Endocrinology, The Christie Hospital NHS Trust, Wilmslow Road, Manchester, M20 9BX, UK

**Keywords:** bone mineral density, acute lymphoblastic leukaemia

## Abstract

Bone mineral density (BMD), serum osteocalcin and type I collagen C-telopeptide (ICTP) were assessed in a cohort of 31 (16 males) adults who had received cranial irradiation in childhood as part of their treatment for acute lymphoblastic leukaemia (ALL). Markers of bone turnover were compared with those of 35 age and body mass index (BMI) matched young adults (18 male). Growth hormone status had previously been determined using an insulin tolerance test and arginine stimulation test. Eight patients were classified as severe growth hormone deficiency (group 1), 12 patients as growth hormone insufficient (group 2) and 11 patients as normal (group 3). Vertebral trabecular BMD, lumbar spine and femoral neck integral BMD and forearm cortical bone mineral content (BMC) was measured 17.8 (6.8–28.6) years after cranial irradiation and was expressed as Z (standard deviation) scores. There was a significant reduction in vertebral trabecular BMD (median Z score –1.25, *P* < 0.001), in lumbar spine integral BMD (median Z score –0.74, *P* = 0.001), in forearm cortical BMC (median Z score –1.35, *P* < 0.001), and less so in femoral neck integral BMD (median Z score –0.43, *P* = 0.03). There was no difference among the growth hormone status groups for the following BMD measurements: vertebral trabecular BMD, lumbar spine integral BMD or femoral neck integral BMD (*P* = 0.8, *P* = 0.96 and *P* = 0.4 respectively). There was only a marginal significant difference for BMD at the wrist between growth hormone status groups (*P* = 0.04). There was no correlation between the BMD measurements with time since or age at diagnosis and no difference in markers of bone turnover between patients and controls; median serum osteocalcin 13.3 and 12.0 ng ml (*P* = 0.7), respectively, and for ICTP 5.0 and 4.9 μg L (*P* = 0.67) respectively. In conclusion, there is a highly significant reduction in BMD in young adults following treatment for ALL in childhood. The reduction in BMD affects both trabecular and cortical bone but did not seem to be related to time since diagnosis, age at diagnosis, or current growth hormone status. Possible explanations include a direct effect of chemotherapy, steroids or both on bone during childhood and hence an effect on the accretion of bone mass. In view of the risk of fractures in patients with osteopenia, adults treated for ALL in childhood may be at an increased risk of bone fractures later in life irrespective of the underlying cause of the osteopenia and thus intervention should be considered. © 1999 Cancer Research Campaign


*British Journal of Cancer* (1999) **79**(11/12), 1859–1863
© 1999 Cancer Research Campaign
Article no. bjoc.1998.0296

### Reduced bone mineral density in young adults following cure of acute lympblastic leukaemia in childhood

**BMD Brennan,[1] A Rahim[3], JA Adams[2], OB Eden[1] and SM Shalet[3]**

[1]Department of Paediatric Oncology, Royal Manchester Children's Hospital, Hospital Road, Manchester M27 4HA, UK; [2]Department of Radiology, University of Manchester, Oxford Road, Manchester M13 9PT, UK; and [3]Department of Endocrinology, The Christie Hospital NHS Trust, Wilmslow Road, Manchester M20 9BX, UK

**Summary** Bone mineral density (BMD), serum osteocalcin and type I collagen C-telopeptide (ICTP) were assessed in a cohort of 31 (16 males) adults who had received cranial irradiation in childhood as part of their treatment for acute lymphoblastic leukaemia (ALL). Markers of bone turnover were compared with those of 35 age and body mass index (BMI) matched young adults (18 male). Growth hormone status had previously been determined using an insulin tolerance test and arginine stimulation test. Eight patients were classified as severe growth hormone deficiency (group 1), 12 patients as growth hormone insufficient (group 2) and 11 patients as normal (group 3). Vertebral trabecular BMD, lumbar spine and femoral neck integral BMD and forearm cortical bone mineral content (BMC) was measured 17.8 (6.8–28.6) years after cranial irradiation and was expressed as Z (standard deviation) scores. There was a significant reduction in vertebral trabecular BMD (median Z score –1.25, $P < 0.001$), in lumbar spine integral BMD (median Z score –0.74, $P = 0.001$), in forearm cortical BMC (median Z score –1.35, $P < 0.001$), and less so in femoral neck integral BMD (median Z score –0.43, $P = 0.03$). There was no difference among the growth hormone status groups for the following BMD measurements: vertebral trabecular BMD, lumbar spine integral BMD or femoral neck integral BMD ($P = 0.8$, $P = 0.96$ and $P = 0.4$ respectively). There was only a marginal significant difference for BMD at the wrist between growth hormone status groups ($P = 0.04$). There was no correlation between the BMD measurements with time since or age at diagnosis and no difference in markers of bone turnover between patients and controls; median serum osteocalcin 13.3 and 12.0 ng ml ($P = 0.7$), respectively, and for ICTP 5.0 and 4.9 µg L ($P = 0.67$) respectively. In conclusion, there is a highly significant reduction in BMD in young adults following treatment for ALL in childhood. The reduction in BMD affects both trabecular and cortical bone but did not seem to be related to time since diagnosis, age at diagnosis, or current growth hormone status. Possible explanations include a direct effect of chemotherapy, steroids or both on bone during childhood and hence an effect on the accretion of bone mass. In view of the risk of fractures in patients with osteopenia, adults treated for ALL in childhood may be at an increased risk of bone fractures later in life irrespective of the underlying cause of the osteopenia and thus intervention should be considered.

**Keywords:** bone mineral density; acute lymphoblastic leukaemia

Children with acute lymphoblastic leukaemia (ALL) with no adverse risk factors now have more than a 70% chance of survival (Chessells et al, 1995). As ALL is the most common form of malignant disease found in children, the numbers of these survivors reaching adulthood are significant and hence the long-term complications of their treatment, which include chemotherapy and radiotherapy, become increasingly important.

We have recently shown that a significant proportion of adults treated with cranial irradiation (XRT) in childhood as part of their treatment for ALL, are severely growth hormone (GH)-deficient and a further proportion are GH-insufficient as a result of radiation-induced hypothalamic damage (Brennan et al, 1998). GH-deficient adults have a reduction in bone mineral density (BMD) irrespective of whether the onset of the GH deficiency is in childhood (O'Halloran et al, 1993) or adulthood (Holmes et al, 1994*b*). Reduction in BMD in children treated for ALL has been documented (Gilsanz et al, 1990; Halton et al, 1996) however, the precise interpretation of these findings in a growing skeleton is difficult unless volumetric measurements of bone mass are performed.

Little is known about bone mass in adults treated for ALL in childhood but there is a suggestion that it is reduced and that GH deficiency appears to be a major factor (Nussey et al, 1994) Thus in order to explore the effect of GH status on BMD in adult survivors of childhood ALL in more detail, we have examined the relationship between BMD, and GH status in 31 adults treated for childhood ALL compared with a group of age- and body mass index (BMI)-matched young adults.

*Received 8 July 1998*
*Revised 20 August 1998*
*Accepted 26 August 1998*

*Correspondence to:* SM Shalet

### MATERIAL AND METHODS

### Patients

Thirty-one adults (16 male) were assessed 6.8–28.6 years (median 17.8 years) after cranial irradiation was administered as part of their treatment for ALL. The median age at diagnosis of ALL was 6.9 years (range 1.7–16 years) and at the time of assessment their median age was 23 years (range 18.8–33 years). They had received chemotherapy for 2 or 3 years after cranial irradiation and this had been completed at least 2 years previously in all cases. They were in first remission, none had received any hormone replacement therapy, they had all achieved final height and progressed through puberty spontaneously. All patients at assessment had either normal oestradiol or testosterone levels and gonadotrophins were within the normal range. No patient had a history of fractures. A review of all patients' notes was performed to obtain details of their chemotherapy and radiotherapy. At assessment, height was measured and converted to standard deviation (s.d.) scores using the standards of Tanner et al, (1965*a*, 1965*b*). The study was approved by the local Ethics Committee in Manchester.

### Treatment

Treatment for ALL was carried out at one oncology centre, Royal Manchester Children's Hospital, over 2–3 years with either UKALL protocols I, II, III, IV, V, VI, VII, VIII, X or Memphis V. UKALL protocols I, II, III, V and VII, were standard regimens consisting of vincristine, prednisolone (total dose 5–5.9 gm $m^2$), L-asparaginase, methotrexate (total dose 1.7–1.92 gm $m^2$) and 6-mercaptopurine. UKALL IV, VI and Memphis V contained the same drugs but these were administered in a pulsed manner. UKALL VIII again included the same drugs but these were administered in a sustained manner and UKALL X was similar to the standard regimes but with the addition of intensification blocks in some, but not all, patients. They had all received between 18 and 25 Gy cranial XRT (11 received 18 Gy) and four of the subjects had also received 24 Gy spinal irradiation.

### Controls

The controls consisted of 35 healthy medical students (18 male). Their median age (range) was 21.6 (21–25) years. None had received any chronic medication within the last 3 years and all underwent a physical examination to exclude any previously undetected pathology. Only osteocalcin and type I collagen cross-linked C-telepeptide assay were measured in the controls.

### Growth hormone status

Each subject underwent assessment of growth hormone status using two provocative agents administered individually on two different mornings. The agents used were intravenous (i.v.) soluble insulin (Actrapid) 0.2 IU kg body weight and 20 gm $m^2$ of i.v. arginine (over 30 min). An i.v. canula was inserted after an overnight fast. Blood samples were taken at time 0 min and then every 30 min for a total of 180 min. The provocative agent was administered at time 0 min. Serum GH was measured in each sample and blood glucose was measured throughout the insulin tolerance test (ITT). All subjects achieved a blood glucose of less than 2.0 mmol l during the ITT. The patient cohort was classified into three GH status groups: (group 1 – GH-deficient, peak GH response to both tests of less than 9 mU ml; group 2–GH insufficient, peak GH response to both tests less than 20 mU ml but one or both responses greater than 9 mU ml; group 3–GH normal, peak GH response to one or both tests greater than 20 mU ml (Brennan et al, 1998).

### Assays

### *Serum growth hormone*

Serum GH levels were measur
d by two-site immunoradiometric assay. The reference preparation used was NIBSC 80/505 and the limit of sensitivity of the growth hormone assay was 1 mU l. The inter-assay coefficients of variation (CV) for the assay were 8.8, 5.5 and 6.5% for mean GH results of 9, 25 and 65 mU l.

### *Osteocalcin*

Serum osteocalcin was measured after an overnight fast, using a two-site immunoradiometric assay (DSL, Webster, TX, USA). All the samples were assayed in a single batch. The sensitivity of this assay was 0.27 ng mL. The intra-assay variability was 4.6%, 2.9% and 1.4% at 2.88, 9.84 and 28.2 ng mL respectively.

### *Type I collagen cross-linked C-telopetide (ICTP) assay*

Serum IcTP was measured using a radioimmunoassay (RIA) (Telepeptide ICTP, Orion Diagnostica). The sensitivity of this assay was 0.5 μg l. The intra- and inter-assay CV was 6.2% and 8% respectively

### *Bone densitometry*

Bone mineral density was estimated in the patients only. Measurements were performed at multiple sites using several techniques.

### *Quantitative computed tomography (QCT)*

Measurement of vertebral trabecular BMD at T12 to L3 was performed using single energy QCT on a GE 9800 general purpose scanner (General Electric, Milwaukee, WI, USA) using a low-dose scanning technique and a liquid dipotassium hydrogen phosphate ($K_2HPO_4$) calibration phantom (Cann and Gerant, 1980). In 28 patients QCT was performed using a Philips SR 4000 CT scanner (Philips Medical Systems, Nederlands BV) and a solid calcium hydroxyapatite reference phantom (Image Analysys Inc., Irvine, CA, USA). Mean trabecular BMD was measured in mg cc of mineral equivalents of either $K_2HPO_4$ in water (n = 4) or calcium hydroxyapatite (n = 28). Precision (CV%) of the technique in our department is 1% in normal subjects and 2.5% in osteoporotic patients (Adams and Adams, 1988; Whitehouse et al, 1990).

### *Dual energy X-ray absorptiometry (DXA)*

Measurement of integral (mixed cortical and trabecular) bone was made in the lumbar spine (L2 to L4) and the right femoral neck using a Lunar DPX-L scanner (Lunar Corporation, Madison, WI, USA) (Callum et al, 1989). Mean BMD was measured in g $cm^{-2}$. Precision of the measurement in our department is 0.5% in the spine and 2.5% in the femoral neck.

### *Single X-ray absorptiometry (SXA)*

Bone density measurement was performed by SXA in the non-dominant forearm using an Osteometer DTX-100 scanner (Roedovre, Denmark). Scanning was performed at the distal site, giving a measurement predominantly of cortical bone. The BMD was measured in g $cm^2$. The precision of BMD measurement by SXA is 1%.

To assess whether the measurements made in each individual were normal or reduced, the values were expressed as Z (s.d.)

 *© Cancer Research Campaign 1999*

**Table 1** Bone mineral density for patients expressed as Z scores in GH status groups. (Severe GHD [1], GH insufficient [2] and normal [3]); data expressed as median (range)

| | Group 1 | | Group 2 | | Group 3 | |
|---|---|---|---|---|---|---|
| QCT | –1.26 | (–2.3–0.8) | –1.1 | (–3.5–0.1) | –1.26 | (–3.1–0.95) |
| DXA spine | –0.5 | (–2.1–1) | –0.75 | (–1.6–0.7) | –0.76 | (–1.8–0.2) |
| DXA femoral neck | –0.61 | (–1.4–0.3) | –0.14 | (–1.3–1.1) | –0.43 | (–1.1–1.65) |
| SXA | –1.1 | (–3.4–0.2) | –2 | (–2.9–0.1) | –1.1 | (–1.9–1.8) |

**Table 2** Correlations between bone mineral density measurements at different sites, and time since or age at diagnosis

| | Age at diagnosis | Time since diagnosis | SXA | QCT | DXA spine |
|---|---|---|---|---|---|
| SXA | $r$ = 0.11 | $r$ = 0.07 | | | |
| | $P$ = 0.57 | $P$ = 0.7 | | | |
| QCT | $r$ = –0.2 | $r$ = 0.25 | $r$ = 0.07 | | |
| | $P$ = 0.93 | $P$ = 0.2 | $P$ = 0.73 | | |
| DXA spine | $r$ = –0.17 | $r$ = 0.19 | $r$ = 0.45 | $r$ = 0.33 | |
| | $P$ = 0.38 | $P$ = 0.33 | $P$ = 0.02 | $P$ = 0.08 | |
| DXA femoral neck | $r$ = 0.09 | $r$ = 0.15 | $r$ = 0.14 | $r$ = 0.53 | $r$ = 0.41 |
| | $P$ = 0.62 | $P$ = 0.42 | $P$ = 0.45 | $P$ = 0.004 | $P$ = 0.03 |

scores derived from a comparison with appropriate normal reference data matched for age and sex (Parfitt, 1990). Reference ranges for QCT were drawn from the normative database of the University of California, San Francisco. For QCT vertebral trabecular bone measured on the GE scanner the data published by Block et al, (1989) were used for women and those of Genant et al, (1983) were used for men. For QCT performed on the Philips CT scanner reference ranges provided by Image Analysis Inc. (Irvine, CA, USA) were used (Faulkner et al, 1993). For DXA measurements the reference ranges provided by the manufacturer (Lunar Corporation, software version 1.3) were used and which have been found to be appropriate for the local population. The Z scores for DXA were weight corrected. Similarly, for SXA the manufacturer's reference data was used (Thomsen et al, 1986).

### Statistical analysis

Statistical analysis was performed using non-parametric tests. Comparisons with age- and sex-matched reference data (results expressed as Z scores) were performed using the Wilcoxon matched-pairs signed rank test. Comparisons of Z scores between the GH status groups of patients were performed using the Kruskal–Wallis test. Comparison between BMD measurements at different sites, and BMD with time since diagnosis or age at diagnosis, are expressed as Spearman's correlation coefficients (r). Comparisons of the markers of bone turnover, osteocalcin and ICTP between patients and controls were performed using the Mann–Whitney *U*-test. A *P*-value of less than 0.05 is considered statistically significant. Statistical analyses apply to all 31 patients except for BMD measurements of the spine when patients who had received spinal irradiation ($n$ = 4) were excluded.

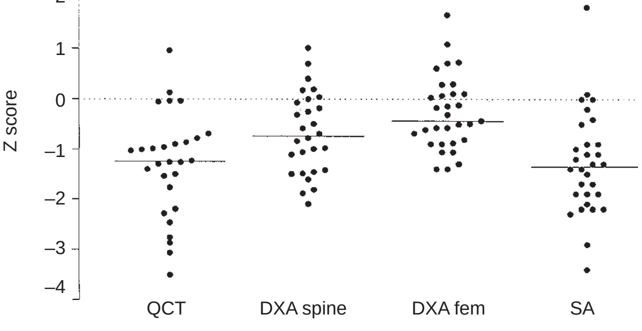

**Figure 1** Bone mineral density Z scores of patients. Horizonal bars represent median values; QCT (–1.25), DXA spine (–0.74), DXA femoral neck (–0.43) and SXA (–1.35)

### RESULTS

There was a highly significant reduction in vertebral trabecular BMD (QCT: median Z score –1.25, range –3.51 to 0.95, $P < 0.001$), in integral bone of the lumbar spine (DXA: median Z score –0.74, range –2.1 to 1.00, $P = 0.001$), in cortical bone of the forearm (SXA: median Z score –1.35, range –3.4 to 1.8, $P < 0.001$), and less so in integral bone of the femoral neck (DXA: median Z score –0.43, range –1.4 to 1.65, $P = 0.03$) [Figure 1].

Height at assessment was analysed only in the 28 subjects who had received cranial irradiation alone (n = 28). The final height SDS was significantly different from zero (median –0.7, range –3.6 to 0.9, $P < 0.001$).

In terms of GH status, eight patients were classified as severe GH-deficient (group 1), 12 patients GH insufficient (group 2) and 11 patients normal (group 3). Bone mineral density data categorized in terms of GH status are summarized in Table 1. There was no difference amongst the GH status groups for the following BMD measurements, QCT spine, DXA spine or DXA femoral neck ($P = 0.8$, $P = 0.96$ and $P = 0.4$ respectively). There was only a marginal significant difference for BMD at the wrist between the

GH status groups ($P$ = 0.04) but the lowest median Z score was in GH status group 2 (median Z score –2) and not the severely GH-deficient group (median Z score – 1.1).

There was no correlation between the BMD measurements at any of the four sites, DXA spine, DXA femoral neck, SXA wrist and QCT spine, with time since or age at diagnosis (Table 2). There were significant correlations between DXA spine with DXA femoral neck and SXA, and also between DXA femoral neck and QCT spine (Table 2).

There was no difference in markers of bone turnover between patients and controls; median (range) serum osteocalcin 13.3 (1.8–40.7) and 12.0 (2.9–43.6) ng ml ($P$ = 0.7), respectively, and for ICTP 5.0 (2.7–8.8) and 4.9 (3.5–8.3) µg L ($P$ = 0.67) respectively.

### DISCUSSION

Height and body size are known to correlate with BMD (Prentice et al, 1994), therefore, we have considered the possibility that the short stature of our patients might be responsible for falsely low BMD estimations. DXA and SA provide areal measurement of BMD and could give falsely low BMD estimations in short subjects. The use of QCT to measure spinal BMD, however, avoids the influence of height on BMD measurement as it is a volumetric measurement of BMD in g $cm^3$. Furthermore, although the final height standard deviation score (SDS) of the whole cohort of patients was significantly reduced (Brennan et al, 1998), the reduction in stature was only noted in a subset of the patients, i.e. severely GH-deficient (group 1), but not in GH status groups 2 or 3. Nonetheless, there was no difference in BMD measurements between the three GH status groups. We conclude that the highly significant decrease in BMD in our patient cohort reflects a significant reduction in bone mass rather than mis-interpretation due to the limitations imposed by certain radiological techniques.

Bone mineral density in children treated for ALL has been investigated before final height has been achieved (Gilsanz et al, 1990; Halton et al, 1996). Gilsanz et al, (1990) examined BMD in 42 children (mean age 11.77 ± 0.53 years) following treatment for ALL using QCT of the spine. The survivors of childhood ALL had lower trabecular bone density in the spine compared with the controls; however, the overall results were heavily influenced by a subgroup of ALL survivors who had received cranial irradiation. Therefore Gilsanz et al (1990) suggested that GH deficiency was a possible cause of the reduction in BMD. Gilsanz et al (1990) also observed no relationship between reduction in BMD and age at time of diagnosis or time since completion of therapy.

Growth hormone deficiency whether of childhood (Kaufman et al, 1992; O’Halloran et al, 1993; De Boer et al, 1994), or adult onset, (Rosen et al, 1993 Holmes et al, 1994*b*) results in a significant reduction in BMD. In adults with childhood onset GH deficiency osteopenia is more severe than adult onset GH deficiency (Holmes et al, 1994*b*), and is likely to be due to failure of accretion of bone mass rather than accelerated bone loss (Kaufman et al, 1992). Growth hormone status in our patients, however, did not seem to influence the reduction in BMD in that patients with severe GH deficiency (group 1), who despite having the greatest loss in height SDS (–2.1) consistent with the presence of GH deficiency for some time (Brennan et al, 1998) did not have the greatest reduction in BMD Z score. This is in contrast to a previous study where GH deficiency appears to be a major factor in the osteopenia seen in a similar cohort of adults survivors of childhood leukaemia (Nussey et al, 1994). The definition of GH deficiency in this latter cohort of patients was, however, less rigorous as it was determined clinically in most patients. GH responses to provocative stimuli were determined in only a minority of patients. Furthermore, their analysis was complicated by including spinal BMD data from patients who had received spinal irradiation a known independent cause of osteopenia (Nussey et al, 1994).

Apart from GH deficiency, there are other possible hormonal influences on BMD. It is well established that hypogonadism in either sex is associated with a reduction in BMD (Stepan et al, 1989; Kreuser et al, 1992; Holmes et al, 1994*a*). However, all patients in this study had progressed through puberty spontaneously and were of normal sex steroid status during adult life when their GH status and BMD were assessed.

Glucocorticoid treatment can cause osteopenia (Baylink, 1983), and as all our patients would have received between 2 and 3 years of prednisolone therapy with a 5-day course every 4 weeks, this must be considered as a possible cause of the osteopenia. Patients with glucocorticoid-induced osteoporosis lose more trabecular than cortical bone; consequently, they have greater bone mineral loss in the vertebrae (Lukert and Raisz, 1990; Reid and Grey, 1993). Furthermore, the predominant result of glucocorticoid treatment is an impairment of bone formation with a decrease in serum osteocalcin, a marker of osteoblastic function (Peretz et al, 1989). In our patient cohort there was a significant reduction in both trabecular and cortical bone with a greater, but not significant, reduction in cortical bone at the wrist. There was no difference between patients and controls in markers of bone turnover, in particular no difference in serum osteocalcin levels.

An alternative aetiology for the osteopenia is cytotoxic chemotherapy. Short-term treatment of rats with methotrexate and doxorubicin has been shown to cause a significant reduction in vertebral trabecular bone volume and in bone formation rate (Friedlaender et al, 1984) raising the possibility that chemotherapy agents may have a direct effect on bone turnover, and hence BMD, in man. Methotrexate is also a key component of the ALL drug treatment received by children at least weekly during the 2 or 3 years of treatment. In childhood ALL treatment regimes where higher doses of prednisolone and methotrexate are used, such as Dana Fabar regime, significant reduction in BMD is seen during therapy (Halton et al, 1996).

In conclusion, we have shown a significant reduction in BMD in adult’s treatment for ALL in childhood. In this cohort current GH status did not seem to influence the reduction in BMD. Possible explanations include a direct effect of chemotherapy, steroids or both on bone during childhood and hence an effect on the accretion of bone mass. This requires further study in patients who are now coming up to adulthood, having been treated for ALL in childhood. The current regimes in the UK have an increased intensity of chemotherapy but less than 10% of children will receive cranial irradiation. It is likely that children who have not received cranial irradiation, have normal GH secretion. The therapeutic implications may differ for these osteopenic young adults dependent upon the primary modalities of therapy that they have been exposed to.

In view of the risk of fractures in patients with osteopenia (Cummings et al, 1993), adults treated for ALL in childhood may be at an increased risk of bone fractures later in life irrespective of the underlying cause of the osteopenia and thus intervention should be considered. If adults treated for ALL in childhood are

*© Cancer Research Campaign 1999*

severely GH-deficient and osteopenic, this is an even stronger reason for GH replacement irrespective of whether or not the osteopenia is due to GH deficiency. In other words the osteopenia of GH deficiency will be superimposed on osteopenia of other causes if left untreated. In those patients osteopenic but not GH-deficient or GH-deficient but unresponsive to GH replacement, drugs which inhibit bone resorption should be considered.

### REFERENCES

Adams JE and Adams PH (1988) The measurement of spinal bone mass by quantitative computed tomography: assessment of precision and different calibration phantoms. *Br J Radiol* **61**: 724

Baylink DJ (1983) Glucocorticoid-induced osteoporosis. *N Engl J Med* **309**: 306–308

Block JE, Smith R, Glueer C-C, Steiger P, Ettinger B and Genant HK (1989) Models of spinal trabecular bone loss as determined by quantitative computed tomography. *J Bone Miner Res* **4**: 249–257

Brennan BMD, Rahim A, Mackie EJ, Eden OB and Shalet SM (1998). Growth hormone status in adults treated for acute lymphoblastic leukaemia in childhood. *Clin Endocrinol* **48**: 777–784

Callum ID, Ell PJ and Ryder JP (1989) X ray dual-photon absorptiometry: a new method for the measurement of bone density. *Br J Radiol* **62**: 587–592

Cann CE and Genant HK (1980) Precise measurement of vertebral mineral content using computed tomography. *J Comput Assist Tomogr* **4**: 493–500

Chessells JM, Bailey C and Richards S (1995) Intensification of treatment and survival in all children with lymphoblastic leukaemia: results of UK Medical Research Council trial UKALL X. *Lancet* **345**: 143–148

Cummings SR, Black DM, Nevitt MC, Browner W, Cauley J, Ensrud K, Genant HK, Palermo L, Scott J and Vogt TM (1993) Bone density at various sites for prediction of hip fractures. *Lancet* **341**: 72–75

De Boer H, Block GJ, Lingen AV, Teule GJJ, Lips P and Van Der Veen EA (1994) Consequences of childhood-onset growth hormone deficiency for adult bone mass. *J Bone Miner Res* **9**: 1319–1327

Faulkner KG, Gluer C-C, Grampp S and Genant HK (1993) Cross-calibration of liquid and solid QCT calibration standards: corrections to the UCSF normative data. *Osteoporosis Int* **3**: 36–42

Friedlaender GE, Tross RB, Doganis AC, Kirkwood JM and Balon R (1984). Effects of chemotherapeutic agents on bone. 1. Short-term methotrexate and doxorubicin (adriamycin) treatment in a rat model. *J Bone Joint Surg* **66A**: 602–607

Genant HK, Cann CE, Pozzi-Mucelli RS and Kanter AS (1983). Vertebral mineral determination by quantitative CT: clinical feasibility and normative data. *J Comput Assist Tomogr* **7**: 554

Gilsanz, V, Carlson ME, Roe TF and Ortega JA (1990) Osteoporosis after cranial irradiation for acute lymphoblastic leukaemia. *J Pediatr* **117**: 238–244

Halton JM, Atkinson SA, Fraher L, Webber C, Gill GJ, Dawson S and Barr RD (1996) Altered mineral metabolism and bone mass in children during treatment for acute lymphoblastic leukaemia. *J Bone Miner Res* **11**: 1774–1783

Holmes SJ, Whitehouse RW, Clark ST, Crowther DC, Adams JE and Shalet SM (1994*a*) Reduced bone mineral density in men following chemotherapy for Hodgkin's disease. *Br J Cancer* **70**: 371–375

Holmes SJ, Economou G, Whitehouse RW, Adams JE and Shalet SM (1994*b*) Reduced bone mineral density in patients with adult onset growth hormone deficiency. *J Clin Endocrinol Metab* **78**: 669–674

Kaufman JM, Taelman P, Vermeulen A and Vandeweghe M (1992) Bone mineral status in growth hormone-deficient males with isolated and multiple pituitary deficiencies of childhood onset. *J Clin Endocrinol Metab* **74**: 118–123

Kreuser ED, Felsenberg D, Behles C, Seibt-Jung H, Mielcarek M, Diehl V, Dahmen E and Thiel E (1992) Long term gonadal dysfunction and its impact on bone mineralization in patients following COPP/ABVD chemotherapy for Hodgkin's disease. *Ann Oncol* **3**: 105–110

Lukert BP and Raisz LG (1990) Glucocorticoid-induced osteoporosis: pathogenesis and management. *Ann Intern Med* **112**: 352–364

Nussey SS, Hyer SL, Brada M and Leiper AD (1994) Bone mineralization after treatment of growth hormone deficiency in survivors of childhood malignancy. *Acta Paediatr Suppl* **399**: 9–14

O'Halloran DJ, Tsatsoulis A, Whitehouse RW, Holmes SJ, Adams JE and Shalet SM (1993) Increased bone density after recombinant human growth hormone (GH) therapy in adults with isolated GH deficiency. *J Clin Endocrinol Metab* **76**: 1344–1348

Parfitt AM (1990) Interpretation of bone densitometry measurements: disadvantages of a percentage scale and a discussion of some alternatives. *J Bone Miner Res* **5**: 537–540

Peretz A, Praet J-P, Bosson D, Rozenberg S and Bourdoux P (1989) Serum osteocalcin in the assessment of corticosteroid induced osteoporosis. Effect of long and short term corticosteroid treatment. *J Rheumatol* **16**: 363–367

Prentice A, Parsons TJ and Cole TJ (1994) Uncritical use of bone mineral density in absorptiometry may lead to size-related artefacts in the identification of bone mineral determinants. *Am J Clin Nutr* **60**: 837–842

Reid IR and Grey AB (1993) Corticosteroid osteoporosis. *Bailliere Clin Rheumatol* **7**: 573–587

Rosen T, Hansson T, Granhed H, Szucs J and Bengtsson B-Å (1983) Reduced bone mineral content in adult patients with growth hormone deficiency. *Acta Endocrinol* **129**: 201–206

Stepan JJ, Lachman M, Zverina J, Pacovsk V and Baylink DJ (1989) Castrated men exhibit bone loss: effect of calcitonin treatment on biochemical indices of bone remodelling. *J Clin Endocrinol Metab* **69**: 523–527

Tanner JM, Whitehouse RH and Takaishi M (1965*a*) Standards from birth to maturity for height, weight, height velocity and weight velocity: British children. *Arch Dis Childhood* **41**: 454–457

Tanner JM, Whitehouse RH and Takaishi M (1965 *b*) Standards from birth to maturity for height, weight, height velocity and weight velocity: British children. *Arch Dis Childhood* **41**: 613–635

Thomsen K, Gotfredsen A and Christiansen C (1986) Is postmenopausal bone loss an age-matched phenomenon? *Calcif Tissue Int* **39**: 123–127

Whitehouse RW, Adams JE, Bancroft K, Vaughan-Williams CA and Elstein M (1990) The effects of nafarelin and danazol on vertebral trabecular bone mass in patients with endometriosis. *Clin Endocrinol* **33**: 365–373

*© Cancer Research Campaign 1999*
